# A Two-Locus Global DNA Barcode for Land Plants: The Coding *rbcL* Gene Complements the Non-Coding *trnH-psbA* Spacer Region

**DOI:** 10.1371/journal.pone.0000508

**Published:** 2007-06-06

**Authors:** W. John Kress, David L. Erickson

**Affiliations:** Department of Botany, National Museum of Natural History, Smithsonian Institution, Washington, D. C., United States of America; Michigan State University, United States of America

## Abstract

**Background:**

A useful DNA barcode requires sufficient sequence variation to distinguish between species and ease of application across a broad range of taxa. Discovery of a DNA barcode for land plants has been limited by intrinsically lower rates of sequence evolution in plant genomes than that observed in animals. This low rate has complicated the trade-off in finding a locus that is universal and readily sequenced and has sufficiently high sequence divergence at the species-level.

**Methodology/Principal Findings:**

Here, a global plant DNA barcode system is evaluated by comparing universal application and degree of sequence divergence for nine putative barcode loci, including coding and non-coding regions, singly and in pairs across a phylogenetically diverse set of 48 genera (two species per genus). No single locus could discriminate among species in a pair in more than 79% of genera, whereas discrimination increased to nearly 88% when the non-coding *trnH-psbA* spacer was paired with one of three coding loci, including *rbcL*. *In silico* trials were conducted in which DNA sequences from GenBank were used to further evaluate the discriminatory power of a subset of these loci. These trials supported the earlier observation that *trnH-psbA* coupled with *rbcL* can correctly identify and discriminate among related species.

**Conclusions/Significance:**

A combination of the non-coding *trnH-psbA* spacer region and a portion of the coding *rbcL* gene is recommended as a two-locus global land plant barcode that provides the necessary universality and species discrimination.

## Introduction

A DNA barcode is an aid to taxonomic identification which uses a standard short genomic region that is universally present in target lineages and has sufficient sequence variation to discriminate among species [1]–[4]. In practice, a DNA sequence from such a standardized gene region can be generated from a small tissue sample taken from an unidentified organism. This sequence is then compared to a library of reference sequences from known species. A match of the sequence from the unknown organism to one of the reference sequences can provide a rapid and reproducible identification. The term “DNA barcode” is used here to refer to a DNA sequence-based identification system that may be constructed of one locus or several loci used together as a complementary unit. DNA barcoding is already emerging as one of the many important tools on the modern taxonomist's work bench despite the debate and controversy among some scientists over the feasibility and utility of genetic identifiers in taxonomic and other applied studies [e.g.], [ 5]–[7]. One factor that is sometimes ignored in this controversy is that the main purpose of DNA barcoding is not to build phylogenetic trees, but to provide rapid and accurate identifications of unidentified organisms whose DNA barcodes have already been registered in a sequence library as described above. Ideally, a barcode should allow unambiguous species identification by having sufficient sequence variation among species and low intraspecific variation. The selection of a barcode locus is, however, complicated by the trade-off that arises between the need for universal application and maximal rates of sequence divergence [8]. Universal application includes standard PCR amplification and sequencing primers as well as the ubiquitous presence of the locus in major land plant lineages. For many groups of animals a segment of the mitochondrial cytochrome c oxidase gene (CO1) has the necessary universality and variability. The 600 bp portion of this gene used as a barcode has sequence divergence among species averaging nearly 11% and provides unambiguous species identification in more than 95% of cases for most of the major animal clades [4], [9]. However, CO1 and other mitochondrial genes have not proven suitable as a barcode for plants because of their low mutation rate and the rapidly changing structure of this genome [10]–[12]. Yet for plants, like animals, DNA barcoding has numerous scientific applications in ecology and evolution as well as direct relevance for more applied fields. A universal land plant barcode is needed, but has yet to be agreed upon [but see 8].

A variety of loci have been suggested as DNA barcodes for plants, including coding genes and non-coding spacers in the nuclear and plastid genomes. For flowering plants the non-coding plastid *trnH-psbA* intergenic spacer region and the multicopy nuclear Internal Transcribed Spacer (ITS) are two of the leading candidates [8]. These two suggested barcodes were demonstrated to be successful in angiosperms and now more extensive trials on non-flowering land plants (mosses, ferns, and gymnosperms) are required to verify their efficacy. The plastid *trnL* intron has been suggested as a possible plant barcode and does have conserved priming sites [13], but the limited interspecific sequence divergence of this region makes it an unlikely universal marker for species-level identification. Six plastid coding regions (*accD, matK, ndhJ, rpoB2, rpoC1,* and *ycf5*) also have been recommended as putative plant barcodes (see http://www.rbgkew.org.uk/barcoding/index.html), but no comparisons of their effectiveness have been published. Finally, even though the plastid *rbcL* gene has been discounted as a species-level discriminator [14]–[15], some researchers have suggested that this region should be included as a standard for comparison to other markers or as a barcode candidate itself [16]–[17]. The advantages of this gene are that it is easily amplified and sequenced in most land plants and it is regarded as a benchmark locus in phylogenetic investigations by providing a reliable placement of a taxon into a plant family and/or genus. However, despite the promise of these regions as putative single-locus barcodes the overall lower levels of mutation rates in plants compared to animals [11] may necessitate a multi-locus barcode to maximally discriminate among plant species [7]–[8].

The objectives of the current study are two-fold: 1) to quantify universal application (PCR and sequencing) and sequence divergence among a phylogenetically diverse set of species pairs for nine putative barcode loci and, 2) to determine which loci, if more than one locus is required, will maximize species identification when combined as a barcode.

## Results

**Figure 1:**
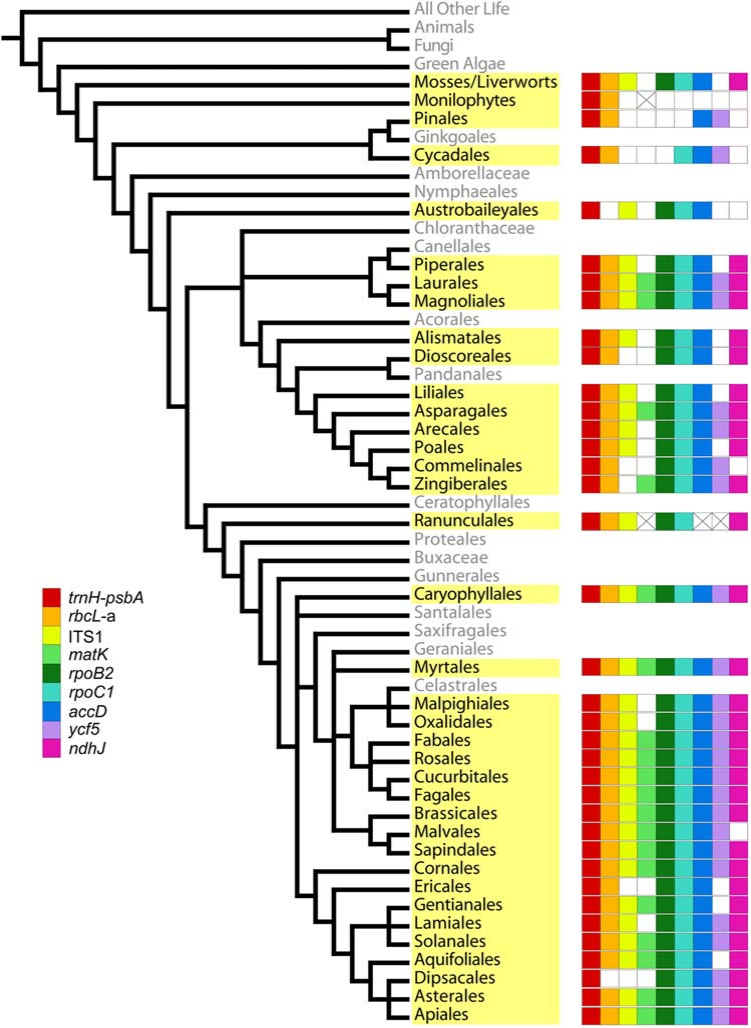
Phylogenetic distribution across land plants of included taxa and PCR success of tested loci. The cladogram indicates the major land plant lineages [34]–[35]. The lineages sampled in this study are highlighted in yellow. The success of each colored-coded primer in amplifying at least one species is indicated for each of the lineages; open white boxes indicate primer failure in all taxa tested; white boxes with an “X” indicate missing sample.

**Figure 2:**
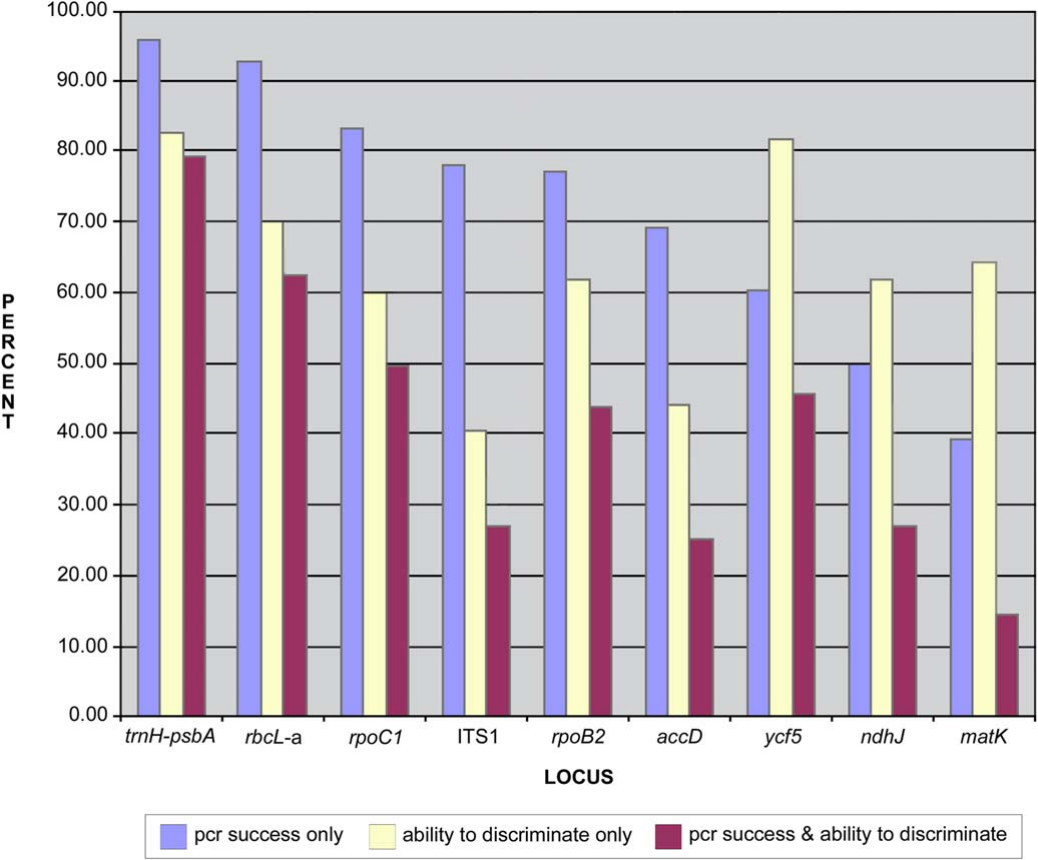
Properties of nine plant loci tested as putative barcodes. Blue bars indicate PCR success; yellow bars indicate percent success in differentiating between species of a pair; maroon bars indicate PCR success combined with the ability to differentiate between species of a pair.

The nine loci varied widely in the universality of their primers and levels of sequence divergence, and hence their potential use as barcodes (Table 1; Figures 1, 2). Only two loci, *trnH-psbA* and *rbcL*-a, exhibited high PCR success with standard primers by amplifying 95.8% (46 of 48 genera) and 92.7% (43 of 48 genera), respectively, of the test species (Figure 1). Three loci, ITS1, *trnH-psbA*, and *rpoB2*, had a mean sequence divergence value greater than two percent while the remaining loci ranged between 0.2% and 1.55% (Tables 1, 2, Figure 2). In the Wilcoxon Signed rank tests ITS1 exhibited a significantly higher degree of divergence (5.7%) than all other loci, followed by *trnH-psbA* (2.69%), which was significantly more divergent than *rpoB2* (2.05%), *rpoC1* (1.38%), and *rbcL*-a (1.29%). Due to the low PCR success of *matK*, and hence the small number of available comparisons, this locus was not shown to be significantly different than any of the other loci, except ITS1. The coding loci *rpoB2*, *rpoC1*, and *rbcL*-a exhibited statistically equal sequence divergence values for the data set (Table 2).

**Table 1:** Comparison of results for nine individual loci tested as putative barcodes on 46–48 species pairs of land plants.

Region	ITS1	*trnH-psbA*	*rbcL*-a	*matK*	*rpoC1*	*ycf5*	*rpoB2*	*ndhJ*	*accD*
**Species pairs tested**	48	48	48	46	48	48	48	47	48
**Mean locus length (bp; standard deviation)**	300 (31.4)	373 (147)	530 (27.5)	501 (18.4)	531 (31.9)	214 (16.8)	485 (15.5)	387 (4)	293 (20.8)
**Percent PCR success**	60.4%	95.8%	92.7%	39.3%	83.3%	50.0%	77.1%	69.1%	78.1%
2 species of pair	27	46	43	14	40	21	34	28	32
1 species of pair	4	0	3	8	3	6	6	9	11
0 species of pair	17	2	2	24	6	21	8	10	5
Angiosperms (80 species)	56	76	74	36	77	47	73	65	72
Gymnosperms (4 species)	0	4	4	0	2	1	0	0	2
Ferns (4 species)	0	4	4	0	2	0	0	0	1
Mosses (8 species)	2	8	8	0	3	0	1	0	1
**Mean percent sequence divergence (n; range; standard deviation)** *	5.7% (27; 14.4–0; 4.58)	2.69% (43; 16.3–0; 3.54)	1.29% (43; 10.1–0; 2.07)	1.13% (14; 14.2–0; 3.76)	1.38% (40; 18–0; 4.14)	1.55% (21; 15.3–0; 3.51)	2.05% (34; 15.0–0; 3.65)	0.20% (28; 2.09–0; 0.527)	1.2% (32; 13.9–0; 1.39)
**Proportion of genera in which species were differentiated (n/n)** **	81.5% (22/27)	82.6% (38/46)	69.8% (30/43)	64.3% (9/14)	60% (24/40)	61.9% (13/21)	61.8% (21/34)	44% (1/28)	40.6% (13/32)
**Total proportion of genera in which species were differentiated (n/n)** ***	45.8% (22/48)	79.1% (34/48)	62.5% (30/48)	14.6% (9/46)	50% (24/48)	27.0% (13/48)	43.8% (21/48)	25.0% (11/44)	27.2% (13/48)

* Mean percent sequence divergence between species pairs across genera that were successfully amplified (n = # of species pairs)

** Proportion of genera in which both species were successfully amplified and exhibited sequence divergence between species (n/n = # of genera in which species of a pair were differentiated/total # of pairs amplified)

*** Proportion of all genera regardless of successful amplification that exhibited sequence divergence between species (n/n = # of genera in which species of a pair were differentiated/total # of pairs sampled)

**Table 2:** Wilcoxon Signed rank tests of divergence among loci.

Locus pairs		Relative ranks	N	P-value	Result
W+	W−				
*trnH-psbA*	*rpoB2*	W+ = 198, W− = 55	22	p< = 0.0211	*trnH-psbA*>*rpoB2*
*trnH-psbA*	*rbcL*-a	W+ = 501, W− = 60	33	p< = 8.466e-05	*trnH-psbA*>*rbcL*-a
*trnH-psbA*	ITS1	W+ = 193, W− = 17	20	p< = 0.0004	*trnH-psbA*≪ITS1
*trnH-psbA*	*rpoC1*	W+ = 293, W− = 53	26	p< = 0.00296	*trnH-psbA*>*rpoC1*
*trnH-psbA*	*matK*	W+ = 26, W− = 40	11	p< = 0.5771	*trnH-psbA* = *matK*
*rbcL*-a	*rpoB2*	W+ = 184.5, W− = 221.50	28	p< = 0.6819	*rbcL*-a = *rpoB2*
*rbcL*-a	ITS1	W+ = 0, W− = 210	20	p< = 1.91e-06	*rbcL*-a≪ITS1
*rbcL*-a	*rpoC1*	W+ = 221, W− = 214	29	p< = 0.9483	*rbcL*-a = *rpoC1*
*rbcL*-a	*matK*	W+ = 38, W− = 28	11	p< = 0.7002	*rbcL*-a = *matK*
*rpoB2*	ITS1	W+ = 5, W− = 185	19	p< = 3.815e-05	*rpoB2*≪ITS1
*rpoB2*	*rpoC1*	W+ = 118, W− = 92	20	p< = 0.6477	*rpoB2* = *rpoC1*
*rpoB2*	*matK*	W+ = 12, W− = 24	8	p< = 0.4609	*rpoB2* = *matK*
*rpoC1*	ITS1	W+ = 0, W− = 171	18	p< = 7.63e-06	*rpoC*≪ITS1
*rpoC1*	*matK*	W+ = 3, W− = 25	7	p< = 0.07812	*rpoC1* = *matK*
ITS1	ITS2	W+ = 75, W− = 16	13	p< = 0.03979	ITS1>ITS2
ITS1	*matK*	W+ = 54, W− = 1	10	p< = 0.003906	ITS1>*matK*

N is the number of genera for which differences in divergence rate were compared, P-value is one sided probability of divergence rates being equal. P-values less than 0.05 were considered significant and interpreted to reflect significant differences in observed rates of divergence.

**Figure 3:**
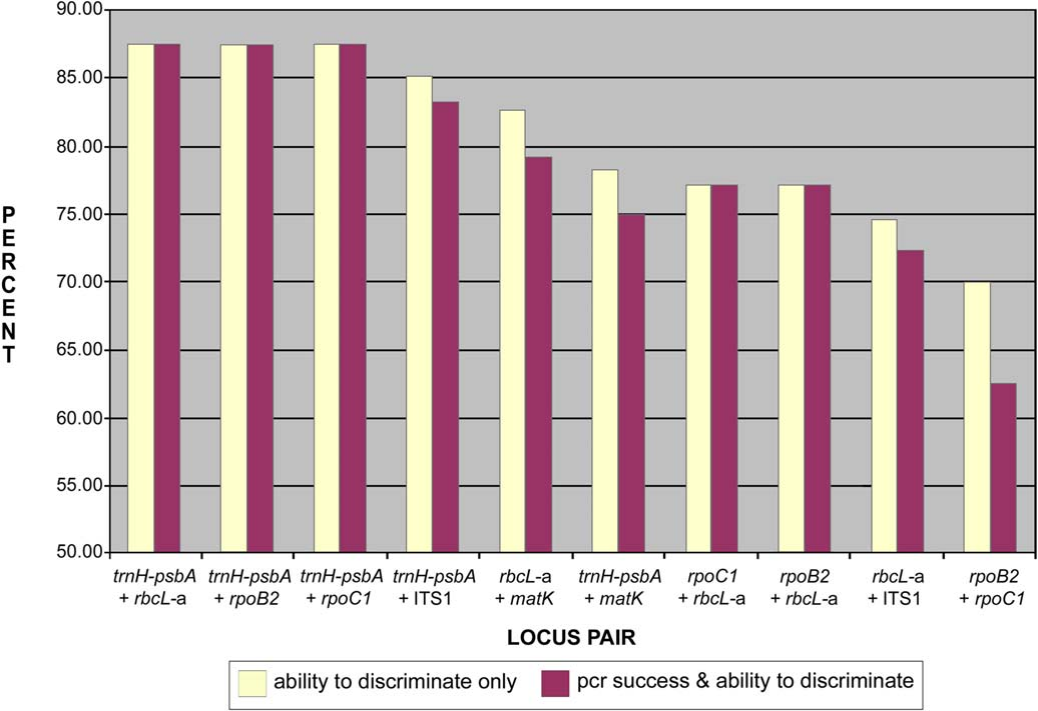
Properties of two-locus pairs tested as putative barcodes. Only those locus pairs with PCR success greater than 90% are included. Yellow bars indicate percent success in differentiating between species of a pair only; maroon bars indicate PCR success combined with the ability to differentiate between species of a pair.

The proportion of genera in which species in a pair could be differentiated also varied widely among loci (Table 1; Figure 3). The *trnH-psbA* spacer and ITS1 showed a much higher level of differentiation (82.6% and 81.5%, respectively) than the other seven loci, none of which had a value higher than 70%. If universal application is incorporated and all genera are considered, then the overall proportion of genera in which species in a pair were differentiated dropped considerably in ITS1 (45.8%) while *trnH-psbA* maintained the highest resolution (79.1%) and *rbcL*-a the second highest (62.5%) with values for all other loci at 50% or less. Six genera were invariant between species in a pair for all of the candidate loci (*Citrus*, *Encephalartos*, *Ludisia*, *Magnolia*, *Raphanus*, and *Sabal*).

The results from data-mining sequences in GenBank, notwithstanding the drawbacks of using such data (e.g., unreliable identifications and uneven sequence quality [18]) and the relatively crude nature of the BLAST search engine, indicated that *trnH-psbA* was successful at returning a correct match. These tests using BLAST were employed as a complement to the primary results on barcode loci derived from the empirical comparative sequence data set. Many of the putative loci had too few sequences in GenBank to conduct a robust test (*accD*, *ndhJ*, *rpoB2*, *rpoC1*, and *ycf5*) or were ruled out due to limitations in universal application (ITS1 and *matK*). For these reasons the *in silico* tests were not exhaustive and only focused on *trnH-psbA* and *rbcL*. Of the 103 genera tested, 75.7% (78 genera) of the searches identified the target sequence as the single best match with the BLASTn search. Similarly *rbcL*, which is a gene noted for its utility as a phylogenetic marker at the rank of family and genus, also demonstrated utility as a species-level identifier in the comparative data-mining tests [17]. Of the original 103 genera tested for *trnH-psbA*, 59 had *rbcL* sequences available in GenBank; of those 59 genera 76.3% (45 genera) of the searches identified the target sequence as the single best match with a BLASTn search (Table 3; Table S1). In the remaining 14 *rbcL* trials in which the correct species was not matched, the search returned more than one species in the correct genus (nine cases) or correct family (five cases). The repeated trials for the *trnH-psbA* spacer with this reduced data set resulted in a slightly higher percentage of success (83.0%) at identification at the species level; the remaining cases identified to the correct genus (Table 3; Table S1). The effect of number of sequences available for a genus in GenBank on the incidence of unique identifications was not statistically significant for either the *trnH-psbA* spacer (t = 1.49; df: 96; P = 0.14) or *rbcL* (t = 1.26; df  = 57; p = 0.21). For the *trnH-psbA* spacer there was also no statistical difference between using partial sequences versus complete sequences in the searches: partial sequences resulted in 28.6% multiple matches while complete sequences resulted in 26.5% multiple matches (Chi-square 0.04; df = 1; p = 0.8).

**Table 3:** GenBank BLASTn results of *trnH-psbA* and *rbcL*-a as a barcode tested singly and as a pair.

	BLAST Results	
**Locus**	**Percentage of single matches to species-level (number of single matches; mean # of sequences/genus; standard deviation)**	**Percentage of single matches to genus/family-level (number of single matches; mean #of sequences/genus; standard deviation)**
***rbcL*** **-a**	76.3% (45; 8.2; 13.6)	23.7% (14; 12.8; 13.8)
***trnH-psbA***	83.0% (49; 19.1; 17.9)	17.0% (10; 19.8; 12.1)
***rbcL*** **-a+** ***trnH-psbA***	95.0% (56; n/a; n/a)	5.0% (3; n/a; n/a)

59 genera, which had sequences available for both loci, were included in the test.

The various combinations of two loci in the multi-locus tests were all more powerful at differentiating between species than either locus individually (Table 4). The *trnH-psbA* spacer when combined with either *rbcL*-a, *rpoB2*, or *rpoC1* demonstrated the highest PCR primer success (100%, i.e., primers amplified for at least one if not both loci across all taxa) and the highest proportion of differentiated species pairs (87.5%; Table 4). The other two-locus combinations that exhibited a proportion of differentiated species pairs better than or equal to the best single locus were *trnH-psbA*+ITS1 (85.1%) and *rbcL*-a+*matK* (82.6%). The PCR success for these two combinations was 99% and 95.8%, respectively. The remaining combinations of loci showed differentiation of species in a pair in less than 82% of the genera.

**Table 4:** Comparisons of results for pairs of two loci for *trnH-psbA*, *rpoB*, *rpoC*, *rbcL*-a, *matK*, and *ITS* tested in all combinations as putative barcodes on 48 species pairs of land plants.

Region	*trnH-psbA*+*rbcL*-a	*trnH-psbA*+*rpoB2*	*trnH-psbA*+*rpoC1*	*trnH-psbA*+ITS1	*trnH-psbA*+*matK*	*rpoB2*+*rbcL*-a	*rpoB2*+*rpoC1*
**Percent PCR success** *	100% (96/96)	100% (96/96)	100% (96/96)	99% (95/96)	95.8% (92/96)	100% (48/48)	90.6% (87/96)
2 species	48	48	48	47	46	48	43
1 species	0	0	0	1	0	0	1
0 species	0	0	0	0	2	0	4
Angiosperms (80)	80	80	80	79	76	80	80
Gymnosperms (4)	4	4	4	4	4	4	0
Ferns (4)	4	4	4	4	4	4	2
Mosses (8)	8	8	8	8	8	8	3
**Proportion of genera in which species were differentiated (n/n)** **	87.5% (42/48)	87.5% (42/48)	87.5% (42/48)	85.1% (40/47)	78.3% (36/46)	77.1% (37/48)	70% (30/43)
**Total proportion of genera in which species were differentiated (n/n)** ***	87.5% (42/48)	87.5% (42/48)	87.5% (42/48)	83.3% (40/48)	75% (36/48)	77.1% (37/48)	62.5% (30/48)
Angiosperms only (n = 40)	85% (34/40)	85% (34/40)	85% (34/40)	82.5% (33/40)	70% (28/40)	72.5% (29/40)	70% (28/40)

Region	*rpoB2*+ITS	*rpoB2*+*matK*	*rpoC1*+*matK*	*rpoC1*+ITS1	*rpoC1*+*rbcL*-a	*rbcL*-a+ITS1	*rbcL*-a+*matK*	ITS1+*matK*
**Percent PCR success** *	83.3% (80/96)	83.3% (80/96)	86.5% (83/96)	89.6% (86/96)	100% (96/96)	100% (96/96)	95.8% (92/96)	70.8% (68/96)
2 species	40	40	40	42	48	48	46	32
1 species	2	0	3	2	0	0	0	4
0 species	6	8	5	4	0	0	0	12
Angiosperms (80)	78	80	78	80	80	80	76	66
Gymnosperms (4)	0	0	0	0	4	4	4	0
Ferns (4)	0	0	2	2	4	4	4	0
Mosses (8)	2	0	3	4	8	8	8	2
**Proportion of genera in which species were differentiated (n/n)** **	80.0% (32/40)	67.5% (27/40)	70.0% (28/40)	78.6% (33/42)	77.1% (37/48)	74.5% (35/47)	82.6% (38/46)	81.3% (26/32)
**Total proportion of genera in which species were differentiated (n/n)** ***	66.7% (32/48)	56.3% (27/48)	58.3% (28/48)	68.8% (33/48)	77.1% (37/48)	72.3% (35/48)	79.2% (38/48)	54.2% (26/48)
Angiosperms only (40 pairs)	80% (32/40)	67.5% (27/40)	65% (26/40)	77.5% (31/40)	72.5% (29/40)	70% (28/40)	75% (30/40)	80% (32/40)

* PCR amplification of either locus for members of a generic pair is regarded as successful amplification for that generic pair

** Proportion of genera in which both species were successfully amplified and exhibited sequence divergence between species (n/n = # of genera in which species of a pair were differentiated/total # of pairs amplified)

*** Proportion of all genera regardless of successful amplification that exhibited sequence divergence between species (n/n = # of genera in which species of a pair were differentiated/total # of pairs sampled)

The results of the GenBank two-locus data-mining tests of *rbcL* and *trnH-psbA* showed that together the two loci provided correct matches at the species level in 95.0% of the trials (Table 3). For the three cases in which the correct species was not matched in the BLASTn search, the query sequence was correctly identified to the appropriate genus.

The differences in the success of discrimination and sequence matching from combining the original sequence data (Table 4) and the BLASTn searches are primarily due to sample size and taxon selection. For the empirical tests (Table 4), taxonomically difficult taxa (e.g., palms, orchids, cycads) were intentionally selected in order to provide a robust test of how well the loci could resolve these species pairs. Whereas the result from the GenBank searches (Table 3) does not necessarily emphasize taxonomically difficult groups and instead reflects more closely the relative abundance of plant families. An increase in sampling of species in the empirical tests that reflects species diversity in nature (e.g., fewer palms and cycads and many more grasses and composites) would likely result in even higher success rates in discriminating between species pairs.

## Discussion

The results suggest that the non-coding *trnH-psbA* intergenic spacer remains the most viable candidate for a single-locus barcode for land plants [8]. In the expanded sampling of loci and taxa the *trnH-psbA* spacer continued to successfully address the trade-off between universal application and high sequence divergence. PCR priming sites within highly conserved flanking coding sequences combined with a non-coding region that exhibits high sequence divergence among species as well as diagnostic insertion/deletion mutations makes the *trnH-psbA* spacer highly suitable as a plant barcode. The significant length variation in *trnH-psbA* due to insertions, deletions, and simple sequence repeats as well as the genomic rearrangement of the inverted repeat in some monocots [19] could be considered as a possible limitation. Non-coding spacers can be difficult to align thereby limiting their utility in phylogenetic studies at higher taxonomic levels [20]. However, this issue has minimal effect on barcoding because the primary goal is species identification and not phylogenetic reconstruction that requires correct alignments. As demonstrated here for *trnH-psbA* GenBank BLASTn searches can find the correct match despite sequence length variation and gaps and thus allow the presence of indels in a target barcode sequence. The local alignment algorithm currently used in a BLASTn search should be improved by substituting a global alignment algorithm, such as the one used in the Barcode of Life Data System (BOLD)[21], that is more efficient at aligning sequences with significant length variation and therefore more successful at matching them within a known sequence database. Search algorithms that use indels as characters should then have greater power to discriminate through exclusion of sequences that do not align and thereby reduce the database population against which the query sequence is compared [22].

The *trnH-psbA* spacer is the most promising single locus for a land plant barcode according to the criteria of universal application and high sequence divergence among species. The intent of the present study was to use these criteria to compare the *trnH-psbA* spacer with other suggested barcode loci across land plants. Several of the plastid genes (*matK, rbcL, rpoB2*, and *rpoC1*) as well as the nuclear ITS region exhibit some features that would make each a possible candidate for a plant barcode (Table 1). However, each of these loci also possesses one or more significant flaws that make it less suitable either due to low PCR amplification success, low levels of sequence divergence, limited utility in non-angiosperms, and/or absence in some land plant lineages. For example, *rpoB2* had a high mean sequence divergence value (2.05%), but poor PCR success in non-angiosperms (failed in all tested gymnosperms, ferns and all but one moss); *rpoC1* had better PCR success (83.3%) than *rpoB2*, but a lower mutation rate (1.38%). The locus *matK*, which has been shown to be quite variable in numerous phylogenetic studies [20], [23], had the lowest amplification success (39.3%) of all loci tested in this study. Further development of primer designs for *matK* and the other loci may improve amplification success, but none of these genes have highly conserved sites near the most variable parts of the locus and hence it is not likely that sufficiently universal primers will be developed. Interestingly, *rbcL*-a in some cases proved better than other coding loci as a barcode. The mean percent sequence divergence for *rbcL*-a ranked sixth, but it exceeded all other loci except ITS1 and *trnH-psbA* in the percent of genera in which species pairs could be differentiated (69.8%). PCR success in *rbcL*-a was also very high (92.7%). ITS1, which was earlier suggested as a possible barcode for flowering plants [8], in this study proved less favorable because of the low primer success across land plants (60.4%). In addition, due to its multicopy nature ITS exhibits high levels of within-species and even within-individual sequence differentiation [24] further reducing its application as a barcode. Three of the tested genes have been shown to be absent in some major groups of land plants, i.e., *accD* absent in grasses, *ndhJ* absent in pines, *ycf5* absent in bryophytes [25], thereby disqualifying them for consideration as widely applicable plant barcodes.

Six of the 48 genera in our sample (*Citrus*, *Encephalartos*, *Ludisia*, *Magnolia*, *Raphanus*, and *Sabal*) were invariant at each of the nine loci in the species pairs tested. Some of these genera are members of families that are known to show low levels of interspecific sequence divergence (e.g., Arecaceae [26], Cycadaceae [27]) and were selected for this reason to be tested in this study. The possible explanations for the lack of sequence variation are several: exceptionally low rates of sequence evolution in these taxa, taxonomic misidentification, and experimental error. If these six genera are examples of overall low rates of sequence divergence, then effective barcoding of such taxa will be difficult no matter which locus is selected. If the lack of sequence variation is due to taxonomic misidentification, i. e., supposedly different species of a pair are actually the same species, or experimental error, i. e., faulty sequencing techniques, a significantly increase in success rate of identification should be possible in the future.

Despite the promise of *trnH-psbA* as a candidate for a land plant barcode, the results reported here suggest that a single locus may not differentiate more than 80% of plant species. If discriminatory power greater than 80% is required, then two or more loci will be needed for maximal species identification in land plants. Here efforts have focused on a two-locus rather than a three or more locus approach because it is simply the most expedient system to use requiring less cost and effort with the desired results. In fact in the present study three-locus systems demonstrated little or no gain over two-locus systems in the proportion of species in a pair that could be differentiated.

A two-locus combinatorial method has been suggested previously [7]–[8], [28], but has never been satisfactorily tested. The results of both generating new test sequences across land plants (Table 4) and in data mining GenBank (Table 3) demonstrate the utility of this approach. The loci chosen should complement each other both in terms of the lineages within which each can discriminate and in balancing type I (incorrect species assignment) and type II (falsely rejecting proper assignment) errors. The combination of the non-coding *trnH-psbA* spacer with one of three coding regions, *rbcL*-a, *rpoB2*, or *rpoC1*, promises the highest universality and the greatest ability to differentiate species pairs in our sample. Complementing a rapidly evolving locus such as the *trnH-psbA* spacer with a more conservative locus (such as the coding locus *rbcL*) can minimize type I errors (such that sequences are robustly assigned to the correct genus at least) and type II errors (higher rates of sequence divergence can discriminate among closely allied species in highly speciose genera). Thus *rbcL* with its proven ease of amplification with broadly applicable primers across land plants and its proven ability to identify taxa at the level of genus and family make it the most appropriate choice for a two-locus barcode coupled with *trnH-psbA*.

The balance of within- and between-species sequence variation is an important aspect of barcode identification [1]–[2], [29] and should be taken into account in the development of a barcode for any group of organisms. Multiple samples per species were not included in the present study to ascertain the level of intraspecific sequence variation for each locus. Such trials are now underway. However, prior reports demonstrate that both *rbcL* [30] and *trnH-psbA* [28] show significantly lower levels of genetic divergence within species than between species.

In conclusion a two-locus barcode that combines a subunit of the coding locus *rbcL* (*rbcL*-a) with the non-coding *trnH-psbA* spacer is recommended. *rbcL*-a provides a strong recognition anchor that will place an unidentified specimen into a family, genus, and sometimes species; the highly variable *trnH-psbA* spacer will further narrow the correct species identification where *rbcL*-a lacks discriminating power, especially in species-rich genera of angiosperms. Both of these loci have standard primers currently available that make them universally amplifiable with the least effort in the broadest range of land plants. This two-locus plant barcode is now being applied to build a library of over 700 species of the world's most important medicinal plants [31; Kress and Erickson, unpubl.]. This barcode library can then be used to test the identity and purity of plant-based medicines and herbals, such as ginseng, ginkgo, echinacea, and St. John's wort, sold in commercial markets and used by consumers. The results of this effort will contribute to the suite of uses of DNA barcodes with substantial economic and social value.

## Materials and Methods

### Tests of a single-locus barcode

Pairs of species from 48 phylogenetically diverse plant genera (of 43 families in 39 orders; Figure 1; Table S2) were compared to quantify levels of interspecific sequence divergence at nine putative barcode loci. The set of taxa includes angiosperms, gymnosperms, ferns, mosses, and liverworts (40 of 48 genera were flowering plants; Figure 1). The selection of plant families and genera for each order was based on availability of tissue samples. The individual species within a genus were chosen without *a priori* expectation of relatedness, hence the congeneric pairs do not necessarily represent nearest neighbor species. Because the experiment was focused on comparing the discriminating power of loci the inclusion of at least some species pairs that could be resolved by all loci increases the statistical power to differentiate among loci. Only a single individual per species was included in the analysis (see comments on intraspecific variation in Discussion). Tissues (leaves for higher plants, thalli for mosses/liverwort) were collected fresh and dried in silica-gel, or recovered from preserved herbarium specimens of various ages; vouchers with institutional accession numbers were prepared for each sample and are stored at the United States National Herbarium at the Smithsonian Institution's National Museum of Natural History. In addition some tissue samples were obtained from the United States Department of Agriculture germplasm resource network and are identified by a discrete USDA accession number (see Table S2).

Uniform DNA extractions were performed on tissue from all species using the DNeasy Plant Mini™ kit (Qiagen, CA). Dry plant material was disrupted in individual lysing tubes with a bead-mill. DNA extraction was conducted following manufacturer's protocols. For all taxa and loci, we conducted PCR amplification in a two stage trial. The first stage used a standard (non-hot-start) DNA polymerase (Biolase™ Taq Polymerase, Bioline) in 25 ul reactions following the protocols of Kress, Wurdack, Zimmer, Weigt and Janzen [8]. The second stage included only samples that did not amplify or that produced multiple PCR products. Samples of both types of failure were re-amplified using a hot-start DNA polymerase (Amplitaq-Gold™ DNA polymerase from Applied Biosystems, CA). The samples that failed to amplify were repeated at lower stringency, (50°C annealing temperatures, and 40 cycles), whereas samples that produced multiple PCR products were repeated at higher stringency (55°C annealing temperatures and 30 cycles). PCR products were then purified for sequencing with ExoSap-IT™ (USB Corp., Ohio) digestion (diluted 4:1 with water) and subsequently used as the template in a 12 µl sequencing reaction. Sequencing reactions were purified by gel flitration with Sephadex G-50 (Amersham Pharmacia Biotech), and then analyzed on an ABI3100 automated sequencer. DNA sequence trace files were aligned with the program Sequencher™ (Gene Codes Corp, MI), and analyzed for levels of sequence divergence as described below. For all loci, alignments between species of a pair were unambiguous and not problematic.

The potential of nine loci as barcodes were compared in this study. The term “locus” is not applied in the strict genetic sense and for convenience refers to both coding and non-coding regions in this discussion. Each of the putative barcodes derived from the seven coding loci represents a subset of the gene that exhibited the highest level of sequence variation and universal amplification within an easily sequenced read length (<700 bp). Six of the loci are described at http://www.rbgkew.org.uk/barcoding/index.html. A 550–600 bp subset of the *rbcL* molecule (termed *rbcL*-a) located at the 5′ end of the large subunit that exhibited maximal sequence variation and universal amplification was also included in the analysis. All available combinations of primers for each of these seven loci were tested on a subset of 4 divergent taxa to select the primer sequences that were subsequently used throughout the experiment (Table S3).

Two spacer regions, one in the nuclear genome (ITS) and one in the plastid genome (*trnH-psbA*), were tested along with the coding loci. The two components of the nuclear internal transcribed spacer (ITS 1 and 2) were compared across 13 of the test genera for size and variability. The ITS1 subset produced a consistently smaller amplicon with fewer artifactual amplification products and exhibited higher levels of sequence divergence relative to ITS2 (Table 2) and was therefore selected for further trials against the other loci. A set of 3 different forward and reverse primers for ITS1 were then evaluated in all possible combinations on the 4 test species, and a consensus primer pair was chosen and applied to the entire taxon set for the empirical experiment. The *trnH-psbA* spacer was treated according to Kress, Wurdack, Zimmer, Weigt and Janzen [8].

Each locus was quantified for PCR amplification success, which is defined as the recovery and successful sequencing of each locus for each species. The phylogenetic range (i.e., major lineages of land plants) over which each locus would amplify with standard procedures was recorded. Two measures of the power of a locus to discriminate among species were calculated: 1) percent sequence divergence between pairs of species and 2) the proportion of the 48 genera for which species in a pair could be differentiated. The first measure was calculated for each barcode by summing the number of mutations separating the two species of a pair relative to the total sequence length, which was then averaged over all genera. The second measure was calculated at two levels: first, for only those genera in which both species amplified and second, for all genera regardless of amplification, thereby incorporating both the level of sequence divergence and universal application into this measure. Significant differences in percent sequence divergence between loci were tested using a Wilcoxon-Signed ranks test contrasting all possible 2-way combinations of loci (*ndhJ*, *accD* and *ycf5* were excluded from these tests for the reasons stated in the Discussion).

### Tests of multi-locus barcodes

The relatively low levels of within-genus sequence divergence suggest that more than one locus may be necessary for species discrimination. Six loci (*trnH-psbA*, ITS, *rbcLa*, *rpoB*, *rpoC1*, and *matK*) were included in contrasts between pairwise combinations were evaluated for their ability to discriminate between species across our sample of land plants. Three coding regions (*accD*, *ycf5*, and *ndhJ*) were eliminated from these tests because they are absent in at least one important group of land plants (see Discussion).

### *In silico* tests of single- and multi-locus barcodes

The sequencing trials of the 48 genera were complemented with data-mining experiments using sequences of candidate barcode loci from GenBank, which is the major repository for sequence data supported by the United States National Center for Biotechnology Information. Although GenBank is not a substitute for a “barcode library,” which will need to be built with high quality DNA sequences from verified voucher specimens, the sequences currently available can provide an independent data set to test the discriminatory powers of various loci. Sufficient sequence records for two of the four most promising loci, *trnH-psbA* and *rbcL*, were available in GenBank whereas accessions for the other two coding loci, *rpoB* and *rpoC*, were insufficient for meaningful comparisons. Sequences for a total of 103 genera (including angiosperms and gymnosperms, but no ferns or mosses) for which six or more full length or partial sequences for *trnH-psbA* were identified and recovered from Genbank. A species sequence representing each genus was used then used as a query sequence in a BLASTn search (short nearly exact search)[32], which is the core search engine available in GenBank for matching sequences. The search returned either a single match (i.e., where the query sequence was returned as the most likely match) or as multiple matches (i.e., where the query sequence plus one or more identical sequences were returned as equally likely). As a comparison, the same set of taxa tested for *trnH-psbA* was used to test the utility of *rbcL* as a complementary locus. Of the 103 genera used in the *trnH-psbA* trials, 59 had corresponding *rbcL* sequences available in GenBank. These 59 genera were queried in the same fashion as above for the portion of *rbcL* and as a repeat trial for the *trnH-psbA* spacer. These 59 genera were then used to test the success of a combined two-locus approach using the BLASTn search. T-tests (for paired samples [33]) were used to determine if the number of sequences available for a genus in GenBank would bias a BLASTn search towards returning a single match versus multiple matches.

## Supporting Information

Table S1 BLASTn trials on 59 genera with both *trnH-psbA* and *rbcL* sequences extracted from GenBank.
(0.09 MB DOC)

Table S2 Taxa sampled in tests of nine putative plant barcode loci.
(0.40 MB DOC)

Table S3 Primer sequences for test loci.
(0.04 MB DOC)
