# Supplementary material for: A Two-Locus Global DNA Barcode for Land Plants: The Coding rbcL Gene Complements the Non-Coding trnH-psbA Spacer Region
Source: PLoS One. 2007 Jun 6;2(6):e508. doi: 10.1371/journal.pone.0000508 (PMC1876818; doi:10.1371/journal.pone.0000508)
Supplement: Table S1 — BLASTn trials on 59 genera with both trnH-psbA and rbcL sequences extracted from GenBank. (0.09 MB DOC) [file pone.0000508.s001.doc]

| **Genus** | **Species** | ***trnH-psbA* match at level of:** | | **Number of species sequences per genus** | | ***rbcL*-a match at level of:** | | **Number of species sequences per genus** |
| --- | --- | --- | --- | --- | --- | --- | --- | --- |
| *Androcymbium* | *ciliolatum* | genus | | 41 | | species | | 7 |
| *Bursera* | *inaguensis* | genus | | 21 | | species | | 1 |
| *Corylopsis* | *pauciflora* | genus | | 15 | | species | | 1 |
| *Cremastosperma* | *cauliflorum* | genus | | 33 | | genus | | 29 |
| *Ephedra* | *viridis* | genus | | 22 | | genus | | 42 |
| *Hedera* | *helix* | genus | | 15 | | genus | | 17 |
| *Hibiscus* | *tiliaceus* | genus | | 7 | | species | | 5 |
| *Menziesia* | *ciliicalyx* | genus | | 8 | | species | | 2 |
| *Saxifraga* | *oppositifolia* | genus | | 5 | | species | | 7 |
| *Symplocos* | *paniculata* | genus | | 31 | | species | | 8 |
|  |  |  | |  | |  | |  |
| *Acacia* | *farnesiana* | species | | 43 | | genus | | 2 |
| *Aconitum* | *lycoctonum* | species | | 7 | | species | | 2 |
| *Actinidia* | *deliciosa* | species | | 10 | | genus | | 35 |
| *Anaxagorea* | *silvatica* | species | | 23 | | species | | 3 |
| *Aucuba* | *japonica* | species | | 5 | | species | | 1 |
| *Brachysiphon* | *acutus* | species | | 14 | | family | | 4 |
| *Canarium* | *ovatum* | species | | 12 | | species | | 1 |
| *Carpinus* | *betulus* | species | | 20 | | species | | 4 |
| *Cinnamomum* | *camphorum* | species | | 5 | | species | | 1 |
| *Cleome* | *viridiflora* | species | | 30 | | species | | 3 |
| *Columnea* | *schiedeana* | species | | 34 | | species | | 2 |
| *Commelina* | *communis* | species | | 1 | | species | | 3 |
| *Dioscorea* | *villosa* | species | | 3 | | species | | 65 |
| *Dryandra* | *foliosissima* | species | | 5 | | species | | 1 |
| *Drymonia* | *serrulata* | species | | 37 | | species | | 1 |
| *Eucalyptus* | *cordata* | species | | 9 | | species | | 1 |
| *Festuca* | *rubra* | species | | 6 | | species | | 3 |
| *Gagea* | *lutea* | species | | 32 | | species | | 2 |
| *Juglans* | *mandshurica* | species | | 17 | | family | | 3 |
| *Klarobelia* | *megalocarpa* | species | | 6 | | family | | 6 |
| *Magnolia* | *coco* | species | | 44 | | species | | 36 |
| *Manglietia* | *conifera* | species | | 3 | | family | | 4 |
| *Manilkara* | *zapota* | species | | 10 | | species | | 2 |
| *Melianthus* | *major* | species | | 8 | | species | | 1 |
| *Mentha* | *longifolia* | species | | 8 | | species | | 3 |
| *Mosannona* | *discolor* | species | | 7 | | species | | 8 |
| *Ostrya* | *virginiana* | species | | 6 | | species | | 2 |
| *Oxandra* | *venezuelana* | species | | 11 | | species | | 11 |
| *Paris* | *fargesii* | species | | 25 | | species | | 2 |
| *Passiflora* | *lutea* | species | | 24 | | species | | 2 |
| *Penaea* | *acutifolia* | species | | 8 | | species | | 3 |
| *Petunia* | *exserta* | species | | 12 | | species | | 1 |
| *Pieris* | *formosa* | species | | 9 | | species | | 9 |
| *Pimpinella* | *saxifraga* | species | | 26 | | species | | 1 |
| *Prunus* | *tomentosa* | species | | 45 | | species | | 38 |
| *Pseudoxandra* | *lucida* | species | | 6 | | genus | | 6 |
| *Rhododendron* | *kaempferi* | species | | 66 | | species | | 3 |
| *Ribes* | *aureum* | species | | 51 | | species | | 1 |
| *Sideroxylon* | *foetidissimum* | species | | 20 | | species | | 2 |
| *Silene* | *atifolia* | species | | 3 | | species | | 5 |
| *Sinojackia* | *rehderiana* | species | | 6 | | family | | 1 |
| *Solanum* | *americanum* | species | | 4 | | species | | 2 |
| *Solidago* | *flexicaulis* | species | | 4 | | genus | | 4 |
| *Taraxacum* | *officinale* | species | | 59 | | species | | 1 |
| *Thymus* | *vulgaris* | species | | 5 | | species | | 2 |
| *Trillium* | *erectum* | species | | 12 | | genus | | 14 |
| *Valeriana* | *urticifolia* | species | | 71 | | species | | 55 |
| *Valerianella* | *locusta* | species | | 15 | | species | | 3 |
| *Viburnum* | *prunifolium* | species | | 43 | | species | | 5 |
|  |  |  |  | |  | |  | |
