# Supplementary material for: A Two-Locus Global DNA Barcode for Land Plants: The Coding rbcL Gene Complements the Non-Coding trnH-psbA Spacer Region
Source: PLoS One. 2007 Jun 6;2(6):e508. doi: 10.1371/journal.pone.0000508 (PMC1876818; doi:10.1371/journal.pone.0000508)
Supplement: Table S2 — Taxa sampled in tests of nine putative plant barcode loci. (0.40 MB DOC) [file pone.0000508.s002.doc]

| **Family** | **Genus** | **Species** | **Voucher*** | **GenBank Accession Numbers** | | | |
| --- | --- | --- | --- | --- | --- | --- | --- |
|  |  |  |  | ***accD*** | **ITS1** | ***ndhJ*** | ***matK*** |
| Amaranthaceae | *Amaranthus* | *hybridus* L. | NMNH Killip 40186 | EF590844 | EF590750 | EF590920 | EF590393 |
| Amaranthaceae | *Amaranthus* | *spinosus* L. | NMNH Wells 4469 | EF590845 | EF590751 | EF590921 | EF590394 |
| Apiaceae | *Angelica* | *archangelica* L. | NMNH Kress 06-8344 | EF590849 | EF590754 | EF590968 | EF590395 |
| Apiaceae | *Angelica* | *dahurica* Maxim. | USDA Ames 26545 | EF590850 | EF590755 |  | EF590396 |
| Aquifoliaceae | *Ilex* | *opaca* Sol. | NMNH Kress 06-8345 | EF590878 | EF590777 | EF590942 | EF590403 |
| Aquifoliaceae | *Ilex* | *paraguariensis* A. St.–Hil. | USDA NA 53625 | EF590879 | EF590778 | EF590943 | EF590404 |
| Araceae | *Amorphophallus* | *titanum* Becc. | NMNH 03-142 | EF590848 |  | EF590924 |  |
| Araceae | *Amorphophallus* | *muelleri* Blume | NMNH-96-216 |  |  | EF590925 |  |
| Arecaceae | *Sabal* | *etonia* Swingle ex Nash. | USBG 000-1065 | EF590912 |  | EF590965 |  |
| Arecaceae | *Sabal* | *minor* (Jacq.) Pers. | USBG 01-1635 | EF590913 |  | EF590964 |  |
| Asclepiadaceae | *Asclepias* | *incarnata* L. | USBG 04-0205 | EF590851 | DQ005967 | EF590926 |  |
| Asclepiadaceae | *Asclepias* | *tuberosa* L. | NMNH Kress 06-8174 | EF590852 | EF590756 | EF590927 |  |
| Asteraceae | *Ambrosia* | *artemisiifolia* L. | NMNH Wells 4495 | EF590846 | EF590752 | EF590922 |  |
| Asteraceae | *Ambrosia* | *trifida* L. | NMNH Wells 30 | EF590847 | EF590753 | EF590923 |  |
| Balsaminaceae | *Impatiens* | *balsamina* L. | USDA OPGC 706 | EF590880 |  |  |  |
| Balsaminaceae | *Impatiens* | *capensis* Meerb. | NMNH Wells 4333a |  |  | EF590973 |  |
| Brassicaceae | *Raphanus* | *raphanistrum* L. | USDA PI271451 | EF590910 |  |  | EF590417 |
| Brassicaceae | *Raphanus* | *sativus* L. | USDA PI508053 | EF590911 |  |  | EF590418 |
| Cactaceae | *Opuntia* | *fragilis*(Nutt.) Haw. | USBG - sn | EF590892 | EF590787 | EF590954 | EF590413 |
| Cactaceae | *Opuntia* | *humifusa* (Raf.) Raf. | NMNH Kress 06-8355 | EF590893 | EF590788 | EF590955 | EF590414 |
| Campanulaceae | *Lobelia* | *cardinalis* L. | USBG 04-0230 | EF590883 |  | EF590946 |  |
| Campanulaceae | *Lobelia* | *inflata* L. | NMNH Kress 06-8352 | EF590884 |  | EF590947 |  |
| Caprifoliaceae | *Viburnum* | *contifolium* D. Don | USDA NA 63028 | EF590918 |  | EF590966 |  |
| Caprifoliaceae | *Viburnum* | *setigerum* Hance | USDA NA 29609 | EF590919 |  | EF590967 |  |
| Commelinaceae | *Commelina* | *imberbis* Ehrenb. ex Hassk. | NMNH 05-014 |  |  |  |  |
| Commelinaceae | *Commelina* | *purpurea* C. B. Clarke | NMNH 94-904 |  |  | EF590971 |  |
| Cucurbitaceae | *Lagenaria* | *sphaerica* E. May | NMNH Kress 06-8346 | EF590881 | EF590779 | EF590944 | EF590405 |
| Cucurbitaceae | *Lagenaria* | *siceraria* (Molina) Standl. | USDA PI 271353 | EF590882 | EF590780 | EF590945 | EF590406 |
| Cyperaceae | *Carex* | *cephalophora* Muhl. ex Willd. | USDA Lea 1483 | EF590855 | EF590758 |  |  |
| Cyperaceae | *Carex* | *oligocarpa* Schkuhr ex Willd. | USDA Lea 1481 | EF590856 | EF590759 |  |  |
| Dicranaceae | *Dicranum* | *flagellare* Hedw. | DUKE Allen 7767 |  |  |  |  |
| Dicranaceae | *Dicranum* | *scoparium* Hedw. | DUKE Allen 21559 |  |  |  |  |
| Dioscoreaceae | *Dioscorea* | *bulbifera* L. | USBG 94-0618 | EF590867 |  |  |  |
| Dioscoreaceae | *Dioscorea* | *villosa* L. | NMNH Beyersdorfer 81 |  |  |  |  |
| Dryopteridaceae | *Dryopteris* | *erythosora* (Eat.) Kuntze | NMNH Kress 06-8357 |  |  |  |  |
| Dryopteridaceae | *Dryopteris* | *goldiana* (Hook. ex Goldie) A. Gray | NMNH Kress 06-8356 |  |  |  |  |
| Fabaceae | *Chamaecrista* | *fasciculate* (Michx.) Greene | USDA DLEG 920271 | EF590857 | EF590760 | EF590970 |  |
| Fabaceae | *Chamaecrista* | *nictitans* (L.) Moench | NMNH Kress 06-8347 | EF590858 | EF590761 |  |  |
| Heliconiaceae | *Heliconia* | *bihai* L. | NMNH RY50 | EF590874 | EF590774 | EF590938 | EF590401 |
| Heliconiaceae | *Heliconia* | *caribaea* Lam. | NMNH Kress 04-7533 | EF590875 |  | EF590939 | EF590402 |
| Hydrangeaceae | *Hydrangea* | *macrophylla* (Thunb.) Ser. | NMNH Kress 06-8351 | EF590876 | EF590775 | EF590940 |  |
| Hydrangeaceae | *Hydrangea* | *xanthoneura* Engl. | NMNH Kress 06-8350 | EF590877 | EF590776 | EF590941 |  |
| Lauraceae | *Cinnamomum* | *camphora* (L.) J.Presl | USDA TARS 4956 | EF590859 |  | EF590928 | EF590397 |
| Lauraceae | *Cinnamomum* | *verum* J. Presl | USBG 98-2670 | EF590860 | EF590762 | EF590929 | EF590398 |
| Magnoliaceae | *Magnolia* | *grandiflora* L. | NMNH Kress 06-8358 | EF590887 |  | EF590950 | EF590407 |
| Magnoliaceae | *Magnolia* | *virginiana* L. | USDA NA 61237 -GU102 | EF590888 |  | EF590951 | EF590408 |
| Malvaceae | *Malva* | *alcea* L. | USDA PI 477991 | EF590889 | EF590783 | EF590974 | EF590409 |
| Malvaceae | *Malva* | *sylvestris* L. | NMNH Kress 06-8168 |  | EF590784 |  | EF590410 |
| Myricaceae | *Morella* | *cerifera* (L.) Small | USDA PI 434149 | EF590890 | EF590785 | EF590952 | EF590411 |
| Myricaceae | *Morella* | *pensylvanica* (Mirb.) Kartesz | USDA NA 66564 BC40 | EF590891 | EF590786 | EF590953 | EF590412 |
| Myrtaceae | *Eucalyptus* | *globulus* Labill. | NMNH 06-8348 | EF590872 | EF590773 |  |  |
| Myrtaceae | *Eucalyptus* | *melliodora* A. Cunn. ex Schauer | NMNH 06-8349 | EF590873 | AF390514 |  |  |
| Orchidaceae | *Dendrobium* | *nobile* Lindl. | USBG 97-0141 | EF590866 | EF590769 | EF590935 |  |
| Orchidaceae | *Dendrobium* | *lindleyi* Steud. | USBG 99-2351 | EF590865 | EF590768 | EF590934 |  |
| Orchidaceae | *Ludisia* | *dawsoniana* (H. Low ex Rchb. f.) Aver. | USBG 88-0018 | EF590885 | EF590781 | EF590948 |  |
| Orchidaceae | *Ludisia* | *discolor* (Ker Gawl.) A. Rich. | USBG 01-0948 | EF590886 | EF590782 | EF590949 |  |
| Oxalidaceae | *Oxalis* | *europeae* Jord. In Schultz | NMNH Wiser 4 | EF590894 |  |  |  |
| Oxalidaceae | *Oxalis* | *violaceae* L. | NMNH 88-364 | EF590895 | EF590789 | EF590975 |  |
| Papaveraceae | *Papaver* | *bracteatum* Lindl. | USDA PI374712 |  | EF590790 | EF590976 | n/a |
| Papaveraceae | *Papaver* | *rhoeas* L. | USDA PI533721 | EF590896 | EF590791 | EF590956 | n/a |
| Passifloraceae | *Passiflora* | *incarnata* L. | USDA_OPGC686 | EF590897 | EF590792 |  |  |
| Passifloraceae | *Passiflora* | *quadrangularis* L. | USDA HPAS30 | EF590898 | EF590793 | EF590957 |  |
| Pinaceae | *Pinus* | *parviflora* Sieb. & Zucc. | NMNH Kressn06-8359 | EF590899 |  |  |  |
| Pinaceae | *Pinus* | *strobus* L. | NMNH Kress 06-8360 | EF590900 |  |  |  |
| Piperaceae | *Piper* | *auritum* Kunth | NMNH 06-8354 | EF590901 | EF590794 | EF590958 |  |
| Piperaceae | *Piper* | *nigrum* L. | USDA TARS 12404 | EF590902 | AF275198 | EF590959 |  |
| Plantaginaceae | *Plantago* | *ovata* Forssk. | USDA PI 596469 | EF590904 | AY101903 | EF590961 |  |
| Plantaginaceae | *Plantago* | *rugelii* Decne. | USDA PI 596469 | EF590903 | EF590795 | EF590960 |  |
| Poaceae | *Calamagrostis* | *divaricata* P.M. Peterson & Soreng | NMNH-sn | EF590853 | EF590757 |  |  |
| Poaceae | *Calamagrostis* | *erectifolia* Hitchc. | NMNH Kress 06-8362 | EF590854 |  | EF590969 |  |
| Poaceae | *Eragrostis* | *pectinaceae* (Michx.) Nees | NMNH Kress 06-8361 | EF590870 | EF590772 | EF590936 |  |
| Poaceae | *Eragrostis* | *tenuiflolia* (A. Rich.) Hochst. ex Steud. | NMNH Kress 06-8363 | EF590871 |  | EF590937 |  |
| Polytrichaceae | *Polytrichum* | *commune* Hedw. | NMNH Eckel 83H5 | EF590905 |  |  |  |
| Polytrichaceae | *Polytrichum* | *juniperinum* Hedw. | NMNH 33767 |  | EF590796 |  |  |
| Porellaceae | *Porella* | *cordaeana* (Hüb.) Moore | DUKE Long 35602 | EF590906 |  |  |  |
| Porellaceae | *Porella* | *densifolia* (Steph.) Hatt. | DUKE Long 28719 | EF590907 |  |  |  |
| Pteridaceae | *Adiantum* | *hispidulum* Sw. | USBG 01-0288 |  |  |  |  |
| Pteridaceae | *Adiantum* | *venustum* D. Don | USBG 00-0037A |  |  |  |  |
| Rosaceae | *Prunus* | *virginiana* L. | NMNH Kress 06-8282 | EF590908 |  | EF590962 | EF590415 |
| Rosaceae | *Prunus* | *yedoense* Matsum. | NMNH Kress 06-8281 | EF590909 |  | EF590963 | EF590416 |
| Rutaceae | *Citrus* | *aurantium* L. | USDA PI 128347 | EF590861 | EF590763 | EF590930 |  |
| Rutaceae | *Citrus* | *reticulata* Blanco | USDA PI 109635 | EF590862 | AM398230 | EF590931 |  |
| Schisandraceae | *Schisandra* | *bicolor* W. C. Cheng | NMNH 06-8343 | EF590914 |  | EF590977 |  |
| Schisandraceae | *Schisandra* | *lancifolia* (Rehder & E. H. Wilson) A. C. Sm. | USDA QLG 062B |  |  |  |  |
| Solanaceae | *Datura* | *meteloides* Dunal | NMNH 06-8150 | EF590864 | EF590766 | EF590972 |  |
| Solanaceae | *Datura* | *stramonium* L. | USBG 06-0525 |  | EF590767 |  |  |
| Sphagnaceae | *Sphagnum* | *platyphyllum* (Lindb. ex Braithw.) Sull. ex Warnst. | NMNH Boles 1644 | EF590915 |  |  |  |
| Sphagnaceae | *Sphagnum* | *contortum* Shultz | NMNH Boles 1738 |  |  |  |  |
| Taccaceae | *Tacca* | *chantrieri* André | NMNH 02-027 | EF590916 |  | EF590978 |  |
| Taccaceae | *Tacca* | *integrifolia* Ker Gawl. | NMNH 02-029 | EF590917 |  | EF590979 |  |
| Taxaceae | *Taxus* | *baccata* L. | NMNH Kress 06-8280 |  |  |  |  |
| Taxaceae | *Taxus* | *cuspidata* Sieb. & Zucc. | NMNH 307-9947 |  |  |  |  |
| Zamiaceae | *Encephalartos* | *ferox* G. Bertol. | USBG 90-00663A | EF590868 | EF590770 |  |  |
| Zamiaceae | *Encephalartos* | *umbeluziensis* R. A. Dyer | USBG 90-0666A | EF590869 | EF590771 |  |  |
| Zingiberaceae | *Curcuma* | *attenuata* Wall. | NMNH 99-127 | EF590863 | EF590764 | EF590932 | EF590399 |
| Zingiberaceae | *Curcuma* | *cochinchinensis* Gagnep. | NMNH 96-207 |  | EF590765 | EF590933 | EF590400 |

*NMNH = National Museum of Natural History, Washington, D.C. USDA = United States Department of Agriculture, Germplasm Resource Centers. USBG = United States Botanic Garden, Washington, D.C. DUKE = Duke University Herbarium, Durham, N.C.

Table S2. (Continued)

| **Family** | **Genus** | **Species** | **GenBank Accession Numbers** | | | | | |
| --- | --- | --- | --- | --- | --- | --- | --- | --- |
|  |  |  | | ***rbcL*-a** | ***rpoB2*** | ***rpoC1*** | ***trnH-psbA*** | ***ycf5*** |
| Amaranthaceae | *Amaranthus* | *hybridus* L. | | EF590495 | EF590419 | EF590583 | DQ006131 | EF590797 |
| Amaranthaceae | *Amaranthus* | *spinosus* L. | | EF590496 | EF590420 | EF590584 | DQ006132 | EF590798 |
| Apiaceae | *Angelica* | *archangelica* L. | | EF590501 | EF590423 | EF590589 | EF590671 |  |
| Apiaceae | *Angelica* | *dahurica* Maxim. | | EF590502 | EF590424 | EF590590 | EF590672 |  |
| Aquifoliaceae | *Ilex* | *opaca* Sol. | | EF590536 | EF590455 | EF590623 | EF590704 |  |
| Aquifoliaceae | *Ilex* | *paraguariensis* A. St.–Hil. | | EF590573 | EF590486 | EF590624 | EF590705 |  |
| Araceae | *Amorphophallus* | *titanum* Becc. | | EF590499 | EF590421 | EF590587 | EF590738 |  |
| Araceae | *Amorphophallus* | *muelleri* Blume | | EF590537 | EF590456 | EF590588 | EF590739 |  |
| Arecaceae | *Sabal* | *etonia* Swingle ex Nash. | | EF590572 | EF590485 | EF590658 | EF590673 | EF590838 |
| Arecaceae | *Sabal* | *minor* (Jacq.) Pers. | | EF590500 | EF590422 | EF590659 | EF590674 | EF590839 |
| Asclepiadaceae | *Asclepias* | *incarnata* L. | | EF590503 | EF590425 | EF590591 |  |  |
| Asclepiadaceae | *Asclepias* | *tuberosa* L. | | EF590504 | EF590426 | EF590592 |  |  |
| Asteraceae | *Ambrosia* | *artemisiifolia* L. | | EF590497 |  | EF590585 | EF590669 | EF590799 |
| Asteraceae | *Ambrosia* | *trifida* L. | | EF590498 |  | EF590586 | EF590670 | EF590800 |
| Balsaminaceae | *Impatiens* | *balsamina* L. | | EF590538 | EF590457 | EF590625 | EF590706 |  |
| Balsaminaceae | *Impatiens* | *capensis* Meerb. | | DQ006071 | EF590458 | EF590626 | DQ006157 |  |
| Brassicaceae | *Raphanus* | *raphanistrum* L. | | EF590570 | EF590483 | EF590656 | EF590736 | EF590836 |
| Brassicaceae | *Raphanus* | *sativus* L. | | EF590571 | EF590484 | EF590657 | EF590737 | EF590837 |
| Cactaceae | *Opuntia* | *fragilis*(Nutt.) Haw. | | EF590551 | EF590471 | EF590639 | EF590717 |  |
| Cactaceae | *Opuntia* | *humifusa* (Raf.) Raf. | | EF590552 | EF590472 | EF590640 | EF590718 | EF590829 |
| Campanulaceae | *Lobelia* | *cardinalis* L. | | EF590541 | EF590461 | EF590629 | EF590709 |  |
| Campanulaceae | *Lobelia* | *inflata* L. | | EF590542 | EF590462 | EF590630 | EF590710 | EF590822 |
| Caprifoliaceae | *Viburnum* | *contifolium* D. Don | |  | EF590491 | EF590665 | EF590748 | EF590842 |
| Caprifoliaceae | *Viburnum* | *setigerum* Hance | |  | EF590492 | EF590666 | EF590749 | EF590843 |
| Commelinaceae | *Commelina* | *imberbis* Ehrenb. ex Hassk. | | EF590513 | EF590437 | EF590603 | EF590681 | EF590807 |
| Commelinaceae | *Commelina* | *purpurea* C. B. Clarke | | EF590514 | EF590438 | EF590604 | EF590682 | EF590808 |
| Cucurbitaceae | *Lagenaria* | *sphaerica* E. May | | EF590539 | EF590459 | EF590627 | EF590707 | EF590820 |
| Cucurbitaceae | *Lagenaria* | *siceraria* (Molina) Standl. | | EF590540 | EF590460 | EF590628 | EF590708 | EF590821 |
| Cyperaceae | *Carex* | *cephalophora* Muhl. ex Willd. | | DQ006089 | EF590429 | EF590595 | DQ006176 |  |
| Cyperaceae | *Carex* | *oligocarpa* Schkuhr ex Willd. | | DQ006090 | EF590430 | EF590596 | DQ006177 |  |
| Dicranaceae | *Dicranum* | *flagellare* Hedw. | | EF590521 |  |  | EF590689 |  |
| Dicranaceae | *Dicranum* | *scoparium* Hedw. | | EF590522 |  |  | EF590690 |  |
| Dioscoreaceae | *Dioscorea* | *bulbifera* L. | | EF590523 | EF590445 | EF590611 | EF590691 |  |
| Dioscoreaceae | *Dioscorea* | *villosa* L. | | DQ006092 | EF590446 | EF590612 | DQ006179 |  |
| Dryopteridaceae | *Dryopteris* | *erythosora* (Eat.) Kuntze | | EF590524 |  |  | EF590692 |  |
| Dryopteridaceae | *Dryopteris* | *goldiana* (Hook. ex Goldie) A. Gray | | EF590525 |  |  | EF590693 |  |
| Fabaceae | *Chamaecrista* | *fasciculate* (Michx.) Greene | | EF590507 | EF590431 | EF590597 | EF590677 | EF590801 |
| Fabaceae | *Chamaecrista* | *nictitans* (L.) Moench | | EF590508 | EF590432 | EF590598 | EF590678 | EF590802 |
| Heliconiaceae | *Heliconia* | *bihai* L. | | EF590532 | EF590451 | EF590619 | EF590700 | EF590817 |
| Heliconiaceae | *Heliconia* | *caribaea* Lam. | | EF590533 | EF590452 | EF590620 | EF590701 | EF590818 |
| Hydrangeaceae | *Hydrangea* | *macrophylla* (Thunb.) Ser. | | EF590534 | EF590453 | EF590621 | EF590702 |  |
| Hydrangeaceae | *Hydrangea* | *xanthoneura* Engl. | | EF590535 | EF590454 | EF590622 | EF590703 | EF590819 |
| Lauraceae | *Cinnamomum* | *camphora* (L.) J.Presl | | EF590509 | EF590433 | EF590599 | EF590750 | EF590803 |
| Lauraceae | *Cinnamomum* | *verum* J. Presl | | EF590510 | EF590434 | EF590600 | EF590751 | EF590804 |
| Magnoliaceae | *Magnolia* | *grandiflora* L. | | EF590545 | EF590465 | EF590633 | EF590711 | EF590823 |
| Magnoliaceae | *Magnolia* | *virginiana* L. | | EF590546 | EF590466 | EF590634 | EF590712 | EF590824 |
| Malvaceae | *Malva* | *alcea* L. | | EF590547 | EF590467 | EF590635 | EF590713 | EF590825 |
| Malvaceae | *Malva* | *sylvestris* L. | | EF590548 | EF590468 | EF590636 | EF590714 | EF590826 |
| Myricaceae | *Morella* | *cerifera* (L.) Small | | EF590549 | EF590469 | EF590637 | EF590715 | EF590827 |
| Myricaceae | *Morella* | *pensylvanica* (Mirb.) Kartesz | | EF590550 | EF590470 | EF590638 | EF590716 | EF590828 |
| Myrtaceae | *Eucalyptus* | *globulus* Labill. | | EF590530 | EF590449 | EF590617 | EF590698 | EF590815 |
| Myrtaceae | *Eucalyptus* | *melliodora* A. Cunn. ex Schauer | | EF590531 | EF590450 | EF590618 | EF590699 | EF590816 |
| Orchidaceae | *Dendrobium* | *nobile* Lindl. | | EF590520 | EF590443 | EF590610 | EF590688 |  |
| Orchidaceae | *Dendrobium* | *lindleyi* Steud. | | EF590519 | EF590444 | EF590609 | EF590687 |  |
| Orchidaceae | *Ludisia* | *dawsoniana* (H. Low ex Rchb. f.) Aver. | | EF590543 | EF590463 | EF590631 |  |  |
| Orchidaceae | *Ludisia* | *discolor* (Ker Gawl.) A. Rich. | | EF590544 | EF590464 | EF590632 |  |  |
| Oxalidaceae | *Oxalis* | *europeae* Jord. In Schultz | | DQ006110 | EF590473 | EF590641 | DQ006207 |  |
| Oxalidaceae | *Oxalis* | *violaceae* L. | | EF590553 | EF590474 | EF590642 | EF590719 |  |
| Papaveraceae | *Papaver* | *bracteatum* Lindl. | | EF590554 | n/a | EF590643 | EF590720 | n/a |
| Papaveraceae | *Papaver* | *rhoeas* L. | | EF590555 | n/a | EF590644 | EF590721 | n/a |
| Passifloraceae | *Passiflora* | *incarnata* L. | | EF590556 | EF590475 | EF590645 | EF590722 | EF590830 |
| Passifloraceae | *Passiflora* | *quadrangularis* L. | | EF590557 | EF590476 | EF590646 | EF590723 | EF590831 |
| Pinaceae | *Pinus* | *parviflora* Sieb. & Zucc. | | EF590558 |  | EF590647 | EF590724 |  |
| Pinaceae | *Pinus* | *strobus* L. | | EF590559 |  | EF590648 | EF590725 |  |
| Piperaceae | *Piper* | *auritum* Kunth | | EF590560 | EF590477 | EF590649 | EF590726 |  |
| Piperaceae | *Piper* | *nigrum* L. | | EF590561 | EF590478 | EF590650 | EF590727 |  |
| Plantaginaceae | *Plantago* | *ovata* Forssk. | | EF590563 | EF590479 | EF590651 | EF590728 | EF590832 |
| Plantaginaceae | *Plantago* | *rugelii* Decne. | | EF590562 | EF590480 | EF590652 | EF590729 | EF590833 |
| Poaceae | *Calamagrostis* | *divaricata* P.M. Peterson & Soreng | | EF590505 | EF590427 | EF590593 | EF590675 |  |
| Poaceae | *Calamagrostis* | *erectifolia* Hitchc. | | EF590506 | EF590428 | EF590594 | EF590676 |  |
| Poaceae | *Eragrostis* | *pectinaceae* (Michx.) Nees | | EF590528 | EF590447 | EF590615 | EF590696 |  |
| Poaceae | *Eragrostis* | *tenuiflolia* (A. Rich.) Hochst. ex Steud. | | EF590529 | EF590448 | EF590616 | EF590697 |  |
| Polytrichaceae | *Polytrichum* | *commune* Hedw. | | EF590564 |  |  | EF590730 |  |
| Polytrichaceae | *Polytrichum* | *juniperinum* Hedw. | | EF590565 |  | EF590653 | EF590731 |  |
| Porellaceae | *Porella* | *cordaeana* (Hüb.) Moore | | EF590566 |  |  | EF590732 |  |
| Porellaceae | *Porella* | *densifolia* (Steph.) Hatt. | | EF590567 |  |  | EF590733 |  |
| Pteridaceae | *Adiantum* | *hispidulum* Sw. | | EF590493 |  | EF590581 | EF590667 |  |
| Pteridaceae | *Adiantum* | *venustum* D. Don | | EF590494 |  | EF590582 | EF590668 |  |
| Rosaceae | *Prunus* | *virginiana* L. | | EF590568 | EF590481 | EF590654 | EF590734 | EF590834 |
| Rosaceae | *Prunus* | *yedoense* Matsum. | | EF590569 | EF590482 | EF590655 | EF590735 | EF590835 |
| Rutaceae | *Citrus* | *aurantium* L. | | EF590511 | EF590435 | EF590601 | EF590679 | EF590805 |
| Rutaceae | *Citrus* | *reticulate* Blanco | | EF590512 | EF590436 | EF590602 | EF590680 | EF590806 |
| Schisandraceae | *Schisandra* | *bicolor* W. C. Cheng | |  | EF590487 |  | EF590740 |  |
| Schisandraceae | *Schisandra* | *lancifolia* (Rehder & E. H. Wilson) A. C. Sm. | | EF590574 | EF590488 | EF590660 | EF590741 |  |
| Solanaceae | *Datura* | *meteloides* Dunal | | EF590517 | EF590441 | EF590607 | EF590685 | EF590811 |
| Solanaceae | *Datura* | *stramonium* L. | | EF590518 | EF590442 | EF590608 | EF590686 | EF590812 |
| Sphagnaceae | *Sphagnum* | *platyphyllum* (Lindb. ex Braithw.) Sull. ex Warnst. | | EF590575 |  | EF590661 | EF590742 |  |
| Sphagnaceae | *Sphagnum* | *contortum* Shultz | | EF590576 |  | EF590662 | EF590743 |  |
| Taccaceae | *Tacca* | *chantrieri* André | | EF590577 | EF590489 | EF590663 | EF590744 | EF590840 |
| Taccaceae | *Tacca* | *integrifolia* Ker Gawl. | | EF590578 | EF590490 | EF590664 | EF590745 | EF590841 |
| Taxaceae | *Taxus* | *baccata* L. | | EF590579 |  |  | EF590746 |  |
| Taxaceae | *Taxus* | *cuspidata* Sieb. & Zucc. | | EF590580 |  |  | EF590747 |  |
| Zamiaceae | *Encephalartos* | *ferox* G. Bertol. | | EF590526 |  | EF590613 | EF590694 | EF590813 |
| Zamiaceae | *Encephalartos* | *umbeluziensis* R. A. Dyer | | EF590527 |  | EF590614 | EF590695 | EF590814 |
| Zingiberaceae | *Curcuma* | *attenuata* Wall. | | EF590515 | EF590439 | EF590605 | EF590683 | EF590809 |
| Zingiberaceae | *Curcuma* | *cochinchinensis* Gagnep. | | EF590516 | EF590440 | EF590606 | EF590684 | EF590810 |

*NMNH = National Museum of Natural History, Washington, D.C. USDA = United States Department of Agriculture, Germplasm Resource Centers. USBG = United States Botanic Garden, Washington, D.C. DUKE = Duke University Herbarium, Durham, N.C.
