# Supplementary material for: A Two-Locus Global DNA Barcode for Land Plants: The Coding rbcL Gene Complements the Non-Coding trnH-psbA Spacer Region
Source: PLoS One. 2007 Jun 6;2(6):e508. doi: 10.1371/journal.pone.0000508 (PMC1876818; doi:10.1371/journal.pone.0000508)
Supplement: Table S3 — Primer sequences for test loci. (0.04 MB DOC) [file pone.0000508.s003.doc]

| Locus | Direction |  | Sequence 5’ – 3’ |
| --- | --- | --- | --- |
| *accD* | 1f |  | AGTATGGGATCCGTAGTAGG |
|  | 4r |  | TCTTTTACCCGCAAATGCAAT |
| *matK* | 2.1f |  | CCTATCCATCTGGAAATCTTAG |
|  | 5r |  | GTTCTAGCACAAGAAAGTCG |
| *ndhJ* | 2f |  | TTGGGCTTCGATTACCAAGG |
|  | 4r |  | TCAATGAGCATCTTGTATTTC |
| *rpoB* | 2f |  | ATGCAACGTCAAGCAGTTCC |
|  | 4r |  | GATCCCAGCATCACAATTCC |
| *rpoC1* | 1f |  | GTGGATACACTTCTTGATAATGG |
|  | 3r |  | TGAGAAAACATAAGTAAACGGGC |
| *ycf5* | 1f |  | GGATTATTAGTCACTCGTTGG |
|  | 4r |  | CCCAATACCATCATACTTAC |
| *rbcL*-a | a_f |  | ATGTCACCACAAACAGAGACTAAAGC |
|  | a_r |  | CTTCTGCTACAAATAAGAATCGATCTC |
| *trnH*-*psbA* | f |  | ACTGCCTTGATCCACTTGGC |
|  | f |  | CGAAGCTCCATCTACAAATGG |
| ITS 1 | 2 |  | CGTAGCTACTTCTTCGCAGC |
|  | 5 |  | CCTTATCATTTAGAGGAAGGAG |
| ITS 2 | 3 |  | TAGCTACTTCTTCGCAGC |
|  | 4 |  | GGTCCAGTCCGCCCTGATGG |
|  |  |  |  |
